# Andrology Legends: A Tribute to Visionaries in Andrology and Reproductive Medicine

**DOI:** 10.5152/tud.2025.251031

**Published:** 2025-12-05

**Authors:** Jonathan Ramsay, Eric Chung

**Affiliations:** 1 The London Clinic, London,UK; 2 Department of Life and Biomedical Sciences, University of Ulster, Belfast, UK; 3 Global Andrology Forum, Global Andrology Foundation, Moreland Hills, Ohio, USA; 4 Department of Urology, University of Queensland/Princess Alexandra Hospital, Brisbane, Australia; 5 Department of Urology, AndroUrology Centre, Brisbane, Australia

## Introduction

The history of andrology is unlike other medical and surgical disciplines; its gestation was from several different ‘stems’ which never quite coalesced. Today, the principal leadership comes from the national and international urological societies, but these have seldom included laboratory-based scientists, embryologists, endocrinologists and gynaecologist, all of whom, broadly and worldwide, have an andrological practice.

The Global Andrology Forum (GAF) is unique as a truly global organization, bringing together members from over 100 countries. It is inclusive and multidisciplinary, uniting basic scientists, embryologists, academic and clinical andrologists, urologists, and gynecologists in a single collaborative platform. As a global forum, GAF reassess contemporary clinical practice and explore new boundaries such as the emerging and rapidly expanding role of artificial intelligence in scientific literature and ethical publication.

The leaders of the GAF may require no introduction to many andrologists and those who well versed in the field of andrology, but their contributions, past and present, deserve some celebration and recognition. In part, this is a fascinating historical record, but in order to develop and to continue to innovate, it behoves all of us to understand how the leaders of the GAF achieved their status.

Fifty years ago, andrology and reproductive medicine barely existed as a specialty. Andrology has only thrived because of the tireless efforts of visionary clinicians and researchers whose dedication has set the standards for scientific rigour, mentorship and patient care. This article, Andrology Legends: a tribute to some of the contemporary visionaries in andrology and reproductive medicine, has been written to honour four such leaders whose work has left a profound and lasting impact on our discipline.

The decision to highlight these distinguished individuals – senior advisers of the non-profit Global Andrology Forum (https://www.globalandrologyforum.com) – stems not only from their outstanding scientific contributions, but also from their enduring commitment to teaching, mentoring and advancing the subject worldwide. The biographical accounts presented here have been compiled from reliable sources and verified records, ensuring both authenticity and accuracy. These narratives are not simply celebrations of achievement, but enduring reflections of a legacy that continues to guide and inspire future generations.

For the reader, whether clinician, scientist, trainee or allied health professional, this article provides more than biographical detail. It offers context and perspective on why these experts matter and how their discoveries have shaped our daily practice, and the lessons that can be drawn from their careers. In understanding the pathways and principles that guided their work, readers gain insight into the values of perseverance, collaboration and innovation that remain critical in advancing patient care and research in male reproductive health.

This article preserves an essential history of andrology and underscores the value of role models in medicine and the need to celebrate excellence, thereby strengthening our community’s commitment to evidence-based practice, scientific progress and holistic care. The stories of these four luminaries remind us that while technologies and therapies evolve, it is the forward thinking vision and scientific integrity of these individuals that continue to define and forward our discipline.

This article is a tribute to the lives and legacies of four remarkable leaders in andrology and reproductive medicine. Each biography not only chronicles individual achievement but also illustrates how dedication, innovation and mentorship have shaped our discipline. Every story is unique, yet together they show how these leaders transformed challenges into opportunities for progress in male reproductive health.

## Biographical Profiles


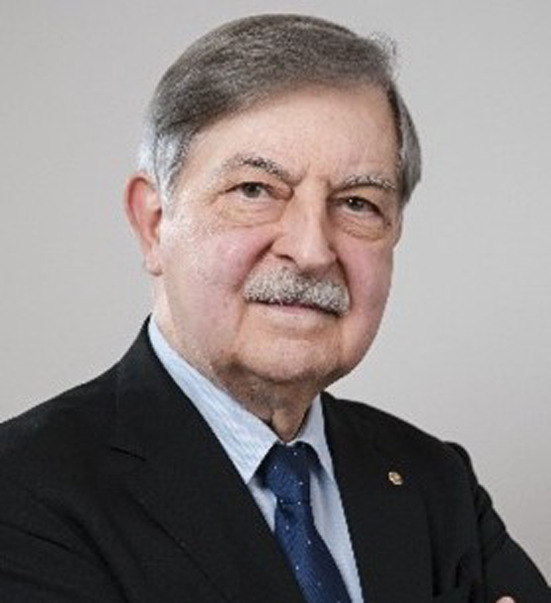


**Giovanni M. Colpi, MD**

## Introduction

Professor Giovanni M. Colpi has been a pioneering leader in andrology, shaping male infertility treatment and microsurgical techniques. His decades-long career has had a lasting global impact through clinical excellence, research, and mentorship.

### Education and Early Career

Born in Milan (Italy), Prof. Colpi trained in urology, andrology, and endocrinology at top Italian universities (Milan, Pisa, Genoa), and earned master’s degrees in clinical sexology (Geneva) and healthcare management (Bocconi, Milan). His early work at Civil Hospital of Magenta advanced ultrasonographic evaluation of male infertility, with his pioneering seminal vesicle study remaining highly cited in andrology.^1^^-^^3^

### Contributions to Male Infertility and Microsurgery

A major milestone in Prof. Colpi’s career was achieving the first full-term pregnancy in an anejaculating spinal cord-injured man using seminal tract washout.^4^ His expertise in microsurgical andrology led to over 2,000 MicroTESE procedures,^5^^-^^7^ microsurgical excisions of testicular incidentalomas,^8^ and numerous microsurgical recanalizations of the seminal tract, helping many infertile men achieve biological fatherhood.

### Academic and Research Contributions

Prof. Colpi has held academic positions as Adjunct Professor of Andrology at the Universities of Milan, Pavia, and L’Aquila for three decades, and has mentored numerous andrologists and reproductive specialists. He has been involved in shaping andrology guidelines, serving on the European Association of Urology’s (EAU) Health Care Office for Male Infertility Guidelines (2000–2008) and the European Academy of Andrology (EAA) Committee for OligoAsthenoTeratozoospermia (2016–2022). His research output includes 173 publications (with an h-index of 34 and over 3,631 citations (Scopus, July 20, 2025).

### Editorial Role

Prof. Colpi has been the Editor-in-Chief of the Reproductive Medicine & Andrology section of *The Journal of Clinical Medicine*, an Editorial Board Member of *Andrologia *and *International Journal of Fertility and Sterility*, and a reviewer for several leading journals.

### Innovation in Surgical Techniques and Patient Care

His surgical innovations have transformed the management of complex andrological conditions. He has pioneered techniques such as artificial spermatocele,^9^ seminal tract washout,^10^ subinguinal sclerotherapy of varicocele with enhanced safety and efficacy,^11^ treatment for obstructive azoospermia due to Müllerian cysts,^12^ and reconstructive procedures for congenital penile curvature.^13^ He has significantly improved fertility outcomes for patients with non-obstructive azoospermia (NOA)^7^ and severe oligoasthenoteratozoospermia (OAT).^14^ He was among the first researchers to investigate the neurophysiological basis of premature ejaculation,^15^ to standardize the use of Doppler ultrasound in varicocele assessment,^16^ and to demonstrate what venous reflux extent measured by Doppler ultrasound is the key predictor of semen improvement after varicocelectomy.^17^ He also promoted perineal muscle rehabilitation as a treatment for erectile dysfunction^18^ and supported penile stretching as a non-invasive approach for “small penis syndrome” and Peyronie’s disease. For years, he has studied epididymal partial obstructions as an often-overlooked cause of OAT^19^ and their ultrasound diagnosis,^20^ recently demonstrating that high untreatable sperm DNA fragmentation in OAT is frequently a sign of epididymal partial obstruction.^21^

Furthermore, he founded Italy’s first sperm bank for cancer patients (1983) and devised the first andrological prevention campaign (1998-2013), screening 11,000 high-school students with his team.

### Commitment to Mentorship and Global Collaboration

Currently serving as Scientific Director and Head of Andrology at Procrea IVF Clinic in Lugano, Switzerland, Prof. Colpi continues to train the next generation of andrologists, offering guidance in both clinical and academic settings. He is also a Senior Advisor to the Global Andrology Foundation (GAF), and a Member of its Management CORE team, contributing to strategic planning, research policy, and global andrological initiatives, and GAF Historian. The “Distinguished GAF Academician” title was awarded to Giovanni in 2024 for his lifelong contributions to Andrology, following a rigorous merit certification process noted for its originality, transparency, and objectivity.^21^

### Legacy and Continuing Impact

Prof. Colpi’s career is a testament to innovation, leadership, and scientific rigor. His work has transformed andrology, enhanced fertility treatments, and paved the way for groundbreaking surgical techniques. His pioneering research and unwavering dedication to patient care have left an indelible mark on andrology, and his ongoing contributions ensure that his legacy will continue to shape the field for years to come.

**Giovanni M. Colpi, MD**

Academician of the European Academy of Andrology

**Distinguished GAF Academician**

Senior Management Advisor and Core Team Member, Global Andrology Forum

Scientific Director of Next Fertility ProCrea, and Head of Andrology Unit, Lugano, Switzerland

Former Head of Uro-Andrology and IVF Department, San Paolo Hospital – University of Milan (Italy)

gmcolpi@yahoo.com


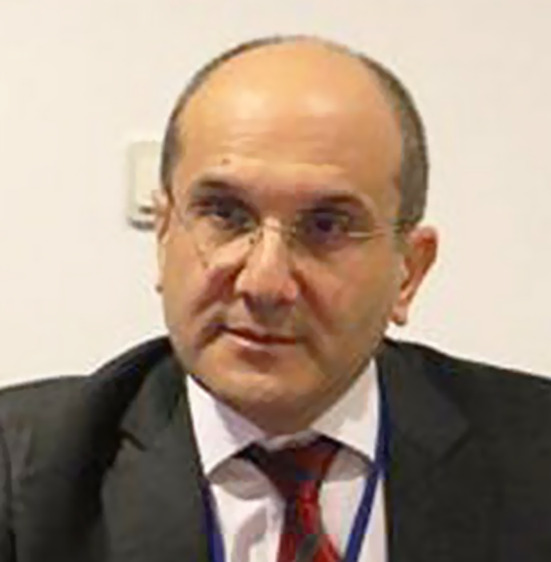


**Professor Ateş Kadıoğlu** is a leading authority in andrology in Türkiye and globally, with over 30 years of contributions to the understanding and treatment of male sexual dysfunction and male reproduction. Beyond his scientific and surgical excellence, he is known for his philosophical and holistic approach to medicine, integrating ethical, ontological, and methodological dimensions. His unique ability to bridge ancient wisdom with modern clinical practice exemplifies the ideal of the wise surgeon scientist—a model that values not only technical skill, but also science, intellectual depth, humanistic sensitivity, and interdisciplinary awareness.

### Education and Specialization

Prof. Kadıoğlu graduated from one of the most prestigious high schools of the country namely Istanbul High School. He graduated from Istanbul University Faculty of Medicine in 1983 and became a urology specialist in 1990. He pursued advanced training in sexual medicine at the University of San Francisco with Prof. Tom Lue and specialized training in male infertility at Baylor College of Medicine in Houston in 1994 with Prof. Larry Lipshultz. He holds the prestigious FECSM (Fellow of the European Committee of Sexual Medicine) title.

### Clinical and Surgical Contributions

Prof. Kadıoğlu is renowned for his expertise in sexual and reproductive medicine surgery, having performed more than 10.000 andrological procedures over 30 years. He has acquired extensive experience in penile prosthesis implantation, with over 2000 cases performed. His proficiency in treating penile curvature (Peyronie’s disease and penile curvature- total ~ 750 cases) through surgical methods has earned him recognition across Europe and Western Eurasia. He also specializes in microsurgical sperm retrieval techniques (Micro-TESE), varicocelectomy (more than 5.000 cases), and surgical treatment of distal ejaculatory duct obstruction.

### Academic and Editorial Contributions

As a Professor of Andrology at Istanbul University, Ateş has published 426 peer-reviewed articles and numerous book chapters to his name, with over 11650 citations and h-factor: 55 (Google Scholar 2025 October). Prof. Kadıoğlu’s academic productivity reflects the breadth and depth of his expertise. His research covers, erectile dysfunction^1^^-^^4^ (Peyronie’s disease^5^^,^^6^ and priapism,^7^ male infertility (varicocele^8^^,^^9^, distal ejaculatory duct obstruction^10^^-^^12^ and female sexual dysfunction.^13^^,^^14^ He has served on the editorial boards of leading journals including *European Urology*, The* Journal of Sexual Medicine*, *Asian Journal of Andrology*, *Andrology*, and *International Urology and Nephrology*, *World Journal of Urology*, *Balkan Medical Journal*, and he is the current editor-in-chief of *Urology Research and Practice*. As a frequent invited speaker at international conferences, Prof. Kadıoğlu has represented Türkiye in global forums and contributed to consensus statements and guideline documents under the European Association of Urology (EAU) and International Society for Sexual Medicine (ISSM), European Society for Sexual Medicine (ESSM) and Global Andrology Forum (GAF).

He has also been actively involved in medical education reform and postgraduate training programs, advocating for evidence-based, patient-centered care in urology. His mentorship has helped cultivate a vibrant community of andrology specialists in the region. He has trained over 100 fellows from Balkan (Kosovo, Albania, Bosnia and Herzegovina, Macedonia), Central Asia (Azerbaijan, Kyrgyzstan, Uzbekistan, Turkmenistan, Kazakhstan) and Middle East.

### Leadership in Professional Societies

Prof. Kadıoğlu has held several prestigious positions in professional societies. He has served as President of the Turkish Andrology Society (2002–2009) and the Turkish Urology Association (2008–2012). He continues to serve as Honorary President of both associations. Internationally, he has held leadership positions within the European Association of Urology (EAU) and has served as a board member of ESAU (2016-2024), where he currently remains an associated board member (2024-2028). He is also a member of the EAU-Sexual and Reproductive Medicine Guideline Committee (2021-). He was the Priapism Committee co-chair of International Consultation of Sexual Medicine in 2010 and 2025. He is a member of the Conflict of Interest (COI) Committee of the International Society for Sexual Medicine (ISSM)(2025-). He chaired major congresses such as Eurasian Andrology Platform (n:10) and Eurasian Urology Platform (n:5) and the Southeastern European Meeting (SEEM) in 2010, European Society for Sexual Medicine (ESSM) in 2014, and Société Internationale d’Urologie (SIU) in 2023. Through these roles, he has influenced policy, research priorities, and clinical practice standards in sexual and reproductive health worldwide.

### Global Impact and Recognition

Prof. Kadıoğlu has been a driving force in establishing clinical standards in male sexual and reproductive health. In 2018, he was named one of the 100 most influential people in Turkish science by Sanko University, published in Turkish Time magazine. In 2021, he received the “Distinguished Career Award” of SIU at the World Urology Congress in Dubai. He is appointed as an honorary Professor by the Ministry of Health of the Republic of Uzbekistan and Eurasian Multidisciplinary University in Tashkent, Uzbekistan. He is appointed as an honorary Professor by the Samarkand State Medical University, Uzbekistan. He is also an honorary Professor of the Kyrgyz Republic, I.K Akhunbaev Kyrgyz State Medical Academy, and the University of Prishtina. He also holds a visiting Professor at Azerbaijan University. He is a member of Senior Advisory Committee of Global Andrology Forum (GAF) since 2024, he was honored with the prestigious “Distinguished GAF Academician” certification, recognizing his lifetime achievements in Andrology through a rigorous, merit-based evaluation process that was original, objective, and transparent.^15^

### Ongoing Legacy

Since 2000, Prof. Ateş Kadıoğlu has led the Department of Andrology at Istanbul University, where he continues to shape the field through surgical excellence, research, and mentorship. His influence extends globally, marked by scientific rigor, compassionate care, and the development of international academic collaborations.

**Ateş Kadioğlu M.D, FECSM**

Chief of Section of Andrology

Istanbul Faculty of Medicine,

**Distinguished GAF Academician**

Senior Management Advisor, Global Andrology Forum

Honorary President of Turkish Society of Andrology

Honorary President of Turkish Association of Urology

Istanbul, Turkey

drkadiogluates@gmail.com


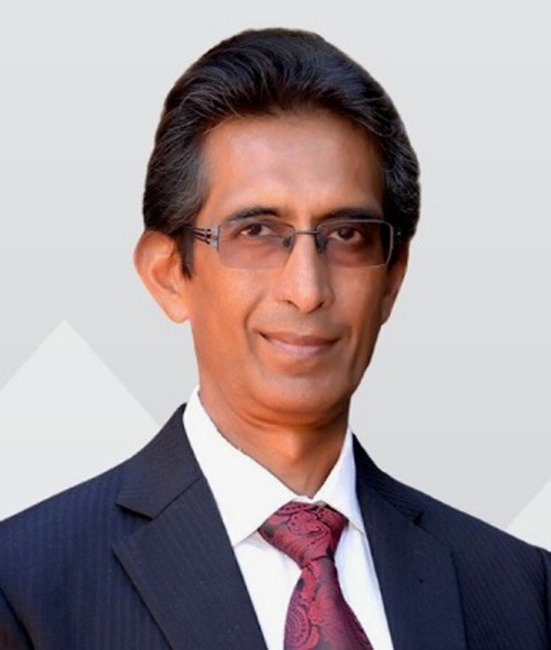


**Dr Rupin Shah,** M.S. (Gen. Surgery); M.Ch.(Urology)

### Introduction

At a time when most doctors in India had not heard of Andrology, Dr Rupin Shah, a qualified urologist, had the vision and the courage to give up general urology and dedicate himself exclusively to the field of male infertility and sexual dysfunction. While taking this difficult decision, he decided that if he were to spend his time exclusively as an andrologist, he would have three goals: a) He would achieve a standard of clinical care in Andrology in India that equaled the best in the world. b) He would innovate to create affordable and locally available therapies and would evolve locally appropriate practice guidelines. c) He would create an in-depth teaching program for urologists so that Andrology develops as a specialty in India.

Over the past 35 years, he has labored hard to fulfill these goals, and through his dedication to the subject, he has changed the practice of Andrology in India.

#### Education and Early Career

He was the first Indian urologist to train abroad, in Belgium in 1986, in Andrological microsurgery, and his pioneering expertise in microsurgical reconstruction for obstructive azoospermia is well known. Over the years, he has been teaching and training other urologists in microsurgical procedures to correct obstructive azoospermia and has conducted operative workshops all over the country and abroad. In 1990, the Urological Society of India (USI) honored his special skills in this area by inviting him to deliver the Society’s Annual State-of-the-Art oration on the topic of “Microsurgery for obstructive azoospermia”.

#### Innovation in Surgical Techniques and Patient Care

A hallmark of his work has been his independent thinking and innovativeness, which led him to reject many Western approaches and develop solutions relevant to his country. When faced with the fact that most of his patients could not afford the imported penile implants, he developed the Shah penile prosthesis – India’s first penile implant.^1^^,^^2^ He then conducted numerous workshops to train other urologists to implant the Shah prosthesis, and today this is the most widely used penile implant in India.

Rejecting the prohibitively priced prostaglandin for intra-penile injection, he pioneered the use of a papaverine-chlorpromazine mixture that cost only a few cents.^3^ This work was awarded the Brij Kishor Patna Prize by the USI, and, thanks to his training workshops, this mixture is now extensively used by urologists in India.

He developed simplified techniques for sperm retrieval for IVF that helped avoid the need for an expensive operating microscope in many cases.^4^ His technique of multiple, atraumatic micro-biopsies was presented at the podium at the annual conference of the American Urological Association in 2004^5^ and has been demonstrated and taught at numerous IVF Conferences in India and abroad.

#### Academic and Research Contributions

Dr Rupin Shah has played a major role in providing Andrology training in India. Initially, he started regular Andrology Training Workshops in Mumbai which were well attended by urologists and sexual medicine physicians. Then, in 1995, he started visiting the well-known Muljibai Patel Urological Hospital in Nadiad to provide andrological training to post-graduate students, and there he started an enormously popular Annual Andrology Training Course that has provided training to thousands of Urologists in India and neighboring countries.

His academic excellence has been honored by the USI with every competitive prize it offers. He has been awarded the CKP Menon Prize for best paper, the Sitaram Memorial Award for best essay, the Brij Kishor Patna Prize for best independent paper, the Vijayawada Best Poster Prize, and the Chandigarh Best Video Prize. He was invited to deliver the prestigious Pinnamaneni Venkateshwar Rao Oration at the Annual Meeting of the USI in 2002, and, in 2007, the USI awarded him the President’s Gold Medal and Oration. Internationally, his new technique for penile revascularization was awarded the President’s Best Video Prize at the 6^th^ World Conference of Impotence in Singapore in 1994.

Dr Rupin Shah has written and published extensively and has a Scopus H-index of 22. He is co-editor of a book on semen analysis^6^ and is currently editing three books on non-obstructive azoospermia, the WHO manual for semen examination, and male sexual dysfunction. He is an associate editor for *Fertility and Reproduction* – the official journal of ASPIRE (Asia Pacific Initiative on Reproduction) and is on the editorial board of *Sexual Medicine Reviews. *He is the Founder-President of the South Asian Society of Sexual Medicine and has held numerous other important international academic positions, including Co-Chair of the International Society of Sexual Medicine (ISSM) Ethics Committee, Co-Chair of the ISSM Scientific Committee, and Co-Chair of the International Committee of Sexual Medicine Guidelines Committee. His dream of establishing Andrology as a sub-specialty in India came to fulfillment a few years ago when the National Board of Examinations of India appointed him Chairman of the committee to formulate the syllabus for a post-doctoral fellowship in Andrology.

All these efforts have earned Dr Rupin Shah the status of father of andrology in India and for this work he was honored with the prestigious Dr B C Roy award by the President of India in 2008. Currently, apart from his clinical work at the Lilavati Hospital & Research Centre and the Sir HN Reliance Hospital in Mumbai, his time is occupied by his role as senior advisor to the Global Andrology Forum (GAF), where he directs the research activities and publications of an international group of over 800 clinicians and researchers. Recognized for his exceptional career in Andrology, Rupin was granted the prestigious “Distinguished GAF Academician” certification in 2024, based on a uniquely structured and impartial merit evaluation method. He has published numerous papers with GAF, including a paper on global practices in varicocele management,^7^ which has been amongst the most widely read papers in the World Journal of Men’s Health, and has recently led an important paper on the state of andrology practice and certification around the globe.^8^

**Dr Rupin Shah,** M.S.(Gen. Surgery); M.Ch.(Urology)

Consultant Andrologist & Microsurgeon,

Lilavati Hospital & Research Centre, Mumbai,

Sir HN Reliance Foundation Hospital, Mumbai

Mumbai, India

**Distinguished GAF Academician**

Senior Management Advisor and Core Team Member, Global Andrology Forum Founder-President, SASSM (South Asian Society of Sexual Medicine)

Co-Chair, International Consultation on Sexual Medicine, 2024

Co-Chair, ISSM Scientific Committee 2024-2026

Co-Chair, Andrology SIG, ASPIRE, 2023-2025

Associate Editor, Fertility & Reproduction journal

Member, Editorial Board, Sexual Medicine Reviews

Member, WHO-ISSM Task Force on Sexual Medicine

Chairman, Post-doctoral Fellowship in Andrology of the National Board of Examinations, India

Email: rupinurvashishah@gmail.com


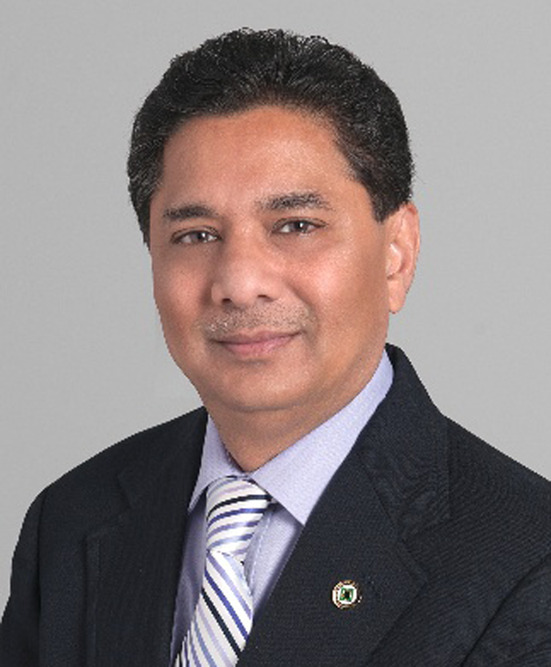


**Ashok Agarwal, PhD, HCLD (ABB), ELD (EMBCOL)**

### Ashok Agarwal – Architect of Global Andrology: From Bench to Worldwide Collaboration: Opening Portrait

Professor Ashok Agarwal has emerged as one of the most influential architects of modern andrology from his work in the last forty years. With over 1,056 peer-reviewed articles (Scopus – October 2025), 225 book chapters, 50 edited medical textbooks, and 18 guest-edited special journal issues, his scientific footprint spans continents and specialties. His work in oxidative stress, sperm DNA fragmentation, proteomics, and fertility preservation has been adopted as reference points for laboratories and clinics worldwide. Recognized by Scopus as the most prolific global author in Male Infertility, Andrology, and Human Assisted Reproduction,^1^^-^^4^ and ranked among the top 2% of scientists globally by Stanford University,^5^ and other sources.^6^^,^^7^ Prof. Agarwal embodies the rare blend of laboratory innovator, researcher-educator, and global research network-builder.

From the bustling academic corridors of Banaras Hindu University (BHU) to the laboratory floors of Harvard Medical School, and eventually to the leadership suites of the Cleveland Clinic, his journey has been driven by an unwavering belief: that excellence in science must be matched by a commitment to mentorship, collaboration, and global accessibility of knowledge.

### Roots and Early Influences

Ashok was born in Lucknow, India, and completed his primary education at the Mahanagar Boy’s High School (Lucknow) in 1969, High School in 1972 from the Central Hindu School (Varanasi) and BSc (Hons) in 1975, M.Sc. (specialization in Reproductive Physiology) in 1977 and a Ph.D. degree in Mammalian Reproductive Biology in 1983 from the Banaras Hindu University (BHU), Varanasi. Under the mentorship of the late Prof. C.J. Dominic in the Department of Zoology, he investigated epididymal structure and biochemical changes across species, work that would sow the seeds for his lifelong interest in male reproductive physiology.

A Rockefeller Foundation Fellowship in Reproductive Biology in 1984 brought him to Harvard Medical School in Boston. He trained under Dr. Anita P. Hoffer in the Division of Urology, Brigham and Women’s Hospital. Here, he undertook seminal studies on gossypol as a male contraceptive and began integrating basic reproductive biology with clinical and translational questions.

### The Harvard Years (1984–1992)

Appointed as Director of Male Infertility Research at Harvard in 1988 and Assistant Professor of Surgery in 1990, Ashok built an early reputation for precision in laboratory andrology and innovation in sperm function testing. Collaborating with urologist Prof. Kevin R. Loughlin at the Brigham and Women’s’ Hospital in Boston, he contributed to new diagnostic paradigms for idiopathic male infertility. These years crystallized his conviction that rigorous methodology must underpin all clinical andrology.

### The Cleveland Clinic Era (1993–2022)

In 1993, he was recruited to the Cleveland Clinic by late Prof. Andrew Novick and late Prof. Anthony J. Thomas, with a mandate to establish its Clinical Andrology and Reproductive Tissue Bank facility as well as develop a research program in male infertility and reproductive physiology. For about 30 years, he transformed these programs into internationally recognized hubs for male infertility diagnostics, advanced sperm function testing, and fertility preservation services, as well as a state-of-the-art center for advanced research on male infertility. His book on andrological testing has become an indispensable resource for thousands of laboratory technologists, clinicians and reproductive professionals engaged in the diagnosis and management of infertile men around the world.^8^ Ashok has the rare honor of seeing four of his research articles cross 1,000 citations mark in Scopus^9^^-^^12^ and four have received over 2,000 citations in Google Scholar.

### Research Highlights

1. **Oxidative Stress and Male Infertility**

Prof. Agarwal’s research established oxidative stress as a central mechanism in male infertility, introducing the ROS–TAC score to quantify oxidative balance and predict fertility potential.^13^ This concept, further elaborated in his books on Male Infertility, and Oxidative Stress,^14^^-^^16^ has influenced antioxidant therapy protocols worldwide.

2. **Sperm DNA Fragmentation**

Through landmark studies and consensus statements, he standardized the TUNEL assay for sperm DNA fragmentation,^17^ guided clinical indications [3], and edited definitive volumes on the subject, including A Clinician’s Guide to Sperm DNA and Chromatin Damage^18^ and has co-edited a special journal issue on this topic.^19^

3. **Proteomics and Biomarker Discovery**

His lab pioneered proteomic mapping of sperm and seminal plasma, identifying biomarkers for varicocele and oxidative stress–related infertility.^20^^-^^22^ These findings, detailed in Proteomics in Human Reproduction,^23^ have opened new avenues for precision diagnostics.

4. **Male Oxidative Stress Infertility (MOSI)**

In 2019, he introduced MOSI as a distinct clinical entity, providing clinicians with a structured approach to diagnosing and treating idiopathic infertility with oxidative etiology.^24^

5. **Fertility Preservation**

In addition to technological advances like NextGen®,^25^ his edited textbooks, The Complete Guide to Male Fertility Preservation^26^ and Manual of Sperm Retrieval and Preparation in Human Assisted Reproduction^27^ serve as reference works for clinicians worldwide.

### Global Andrology Forum (2022 – Present)

In 2021, Prof. Agarwal founded the Global Andrology Forum (GAF), now comprising over 850 members from 95 countries, with 14 research teams producing consensus guidelines, systematic reviews, and educational initiatives.^28^^-^^30^ GAF’s unique mentorship-based training model and global surveys have helped shape emerging clinical guidelines, filling gaps in existing literature. In the last 3 years of its existence, GAF researchers have published over 100 research articles in high-impact journals, edited six medical textbooks,^31^^-^^33^ and 3 special journal issues. GAF’s merit-based academic certification program^34^ is a first in the field, ensuring recognition based solely on transparent achievement metrics.

### Mentorship, Education, and Training Legacy

A hallmark of his career has been the training of more than 525 scientists and clinicians from over 55 countries. His annual Summer Internship in Reproductive Medicine at the Cleveland Clinic — blending laboratory skills, biostatistics, scientific writing, and communication — ran for over 12 years and earned multiple Scholarships in Teaching Awards from the Case Western Reserve University. His ART hands-on training programs have trained more than 200 embryologists from 44 countries.

During the COVID-19 pandemic, Ashok pivoted to virtual education, delivering webinars and scientific-writing workshops^35^ that trained over 10,000 participants in 105 countries.

### Recognition and Enduring Influence

Prof. Agarwal’s global influence has been recognized with numerous awards, honors and accolades. He serves on editorial boards of several journals and is dedicated to elevating the Global Andrology Foundation to a new era of worldwide influence and scientific leadership.

### Conclusion: The Continuing Arc

As the Founder and the Director of Research of GAF, Prof. Agarwal continues to lead initiatives that set benchmarks in male infertility and sexual medicine. GAF’s mission is to produce highest quality research, particularly systematic reviews and meta-analyses, global surveys and clinical guidelines on contentious topics, while integrating basic science with clinical practice. Through scientific meetings, training programs, and mentorship, GAF fosters global collaboration and advances evidence-based care that respects diverse healthcare contexts and patient values. Prof. Agarwal’s career exemplifies how scientific rigor, coupled with a culture of mentorship and collaboration, can reshape an entire discipline. His philosophy is simple yet profound: excellence is not an act, but a habit. It begins with the smallest of tasks, where meticulous effort builds into monumental achievements. In his words, “the difference between ordinary and extraordinary is that little extra—so why ever settle for less?”.

**Ashok Agarwal, PhD, HCLD, ELD (ABB)**

Founder and Director, Global Andrology Forum

President, Global Andrology Foundation

Emeritus Staff, Cleveland Clinic Foundation

Professor of Urology, Cleveland Clinic Lerner College of Medicine of Case Western Reserve University

Certified High-Complexity Clinical Lab Director in Andrology (HCLD-ABB)Associate Board Member, European Section of Andrological Urology, ESAU

Former Director, Andrology Laboratory and Reproductive Tissue Bank and the American Center for Reproductive Medicine, Cleveland Clinic

Cleveland, Ohio, USA

Email: agarwaa32099@outlook.com

### Conclusion

Collectively, the contributions of these highly distinguished experts form a rich mosaic for progressive andrology—spanning surgery, research, education, and international collaboration. Beyond their individual accomplishments, their shared legacy emphasizes clinical and research excellence, tireless mentorship, and scientific innovation as guiding principles for future generations. It is on this foundation that we now offer our concluding reflections.

As we reflect on the careers and contributions of these pioneers, we are reminded that progress in medicine is not only measured in data and publications but in the lives improved, the “students” inspired, and the communities transformed. Paying tribute to these legends serves as both acknowledgment of their remarkable journeys and a call to action for all of us to emulate their achievements and be the best that one can be. Their legacies illuminate the path forward for andrology, ensuring that the field continues to thrive, innovate, and deliver compassionate best-evidenced healthcare practice.

## References

[1] Colpi GM Ballerini G Zanollo A. Ultrasonography of the seminal vesicles in infertility. Progr. Reprod. Biol. Med. 12:124 142. Basel: Karger; 1985.

[2] Colpi GM Casella F Zanollo A Functional voiding disturbances of the ampullo-vesicular seminal tract: a cause of male infertility. Acta Eur Fertil. 1987;18(3):165 179. 3125711

[3] Colpi GM Negri L Mariani M Balerna M. Semen anomalies due to voiding defects of the ampullo-vesicular tract. Infertility due to ampullo-vesicular voiding defects. Andrologia. 1990;22(Suppl 1):206 218. (doi: 10.1111/j.1439-0272.1990.tb02086.x) 2132071

[4] Colpi GM Negri L Stamm J Balerna M. Full-term pregnancy obtained with sperm recovered by seminal tract washout from an anejaculating, spinal cord injury man. J Urol. 1992;148(4):1266 1267. (doi: 10.1016/s0022-5347(17)36886-6) 1404655

[5] Colpi GM Colpi EM Piediferro G Microsurgical TESE versus conventional TESE for ICSI in non-obstructive azoospermia: a randomized controlled study. Reprod Biomed Online. 2009;18(3):315 319. (doi: 10.1016/s1472-6483(10)60087-9) 19298728

[6] Esteves SC Ramasamy R Colpi GM Carvalho JF Schlegel PN. Sperm retrieval rates by micro-TESE versus conventional TESE in men with non-obstructive azoospermia-the assumption of independence in effect sizes might lead to misleading conclusions. Hum Reprod Update. 2020;26(4):603 605. (doi: 10.1093/humupd/dmaa006) 32436569

[7] Colpi GM Caroppo E. Performing microdissection testicular sperm extraction: surgical pearls from a high-volume infertility center. J Clin Med. 2021;10(19):4296. (doi: 10.3390/jcm10194296) PMC8509819 34640310

[8] Colpi GM Carmignani L Nerva F Guido P Gadda F Castiglioni F. Testicular-sparing microsurgery for suspected testicular masses. BJU Int. 2005;96(1):67 69. (doi: 10.1111/j.1464-410X.2005.05569.x) 15963123

[9] Colpi GM Zanollo A Langè A Farina U Beretta G. Artificial spermatocele inserted onto the vas deferens: a clinical report. Acta Eur Fertil. 1983;14(3):203 208. 6367333

[10] Colpi GM Negri L Scroppo FI Grugnetti C Patrizio P. Seminal tract washout: a new diagnostic tool in complicated cases of male infertility. J Androl. 1994;15 suppl:17S 22S. (doi: 10.1002/j.1939-4640.1994.tb01698.x) 7721670

[11] Colpi GM Carmignani L Nerva F Surgical treatment of varicocele by a subinguinal approach combined with antegrade intraoperative sclerotherapy of venous vessels. BJU Int. 2006;97(1):142 145. (doi: 10.1111/j.1464-410X.2006.05915.x) 16336345

[12] Colpi GM Negri L Scroppo FI Grugnetti C. Ultrasonically guided treatment of anomalies of uroseminal carrefour. In: Colpi GM , Balerna M , eds. Treating Male Infertility: New Possibilities. Basel: Karger; 1994:187 198.

[13] Colpi GM Piediferro G Castiglioni F Contalbi G Carmignani L. Penile septoplasty for congenital ventral penile curvature: results in 51 patients. J Urol. 2009;182(4):1489 1494. (doi: 10.1016/j.juro.2009.06.059) 19683765

[14] Baldi E Colpi GM Huang Z-W High sperm DNA fragmentation - finding a needle in the haystack: tips on selecting the best sperm for ICSI and ART. Asian J Androl. 2025;27(2):139 143. (doi: 10.4103/aja202451) 39224976 PMC11949445

[15] Fanciullacci F Colpi GM Beretta G Zanollo A. Cortical evoked potentials in subjects with true premature ejaculation. Andrologia. 1988;20(4):326 330. 3195725

[16] Annoni F Colpi GM Marincola FM Negri L. Doppler examination in varicocele. A standard method of evaluation. J Androl. 1988;9(4):248 252. (doi: 10.1002/j.1939-4640.1988.tb01046.x) 3053549

[17] Cavallini G Scroppo FI Colpi GM. The clinical usefulness of a novel grading system for varicocoeles using duplex Doppler ultrasound examination based on postsurgical modifications of seminal parameters. Andrology. 2019;7(1):62 68. (doi: 10.1111/andr.12556) 30354030

[18] Colpi GM Negri L Nappi RE Chinea B. Perineal floor efficiency in sexually potent and impotent men. Int J Impot Res. 1999;11(3):153 157. (doi: 10.1038/sj.ijir.3900413) 10404284

[19] Colpi GM Caroppo E. Partial Epididymal Obstruction as a Cause of Idiopathic Oligozoospermia: a Reproductive Urologist's Perspective following 35 years of Surgical and Clinical Experience. J Clin Med. 2024;13(2):382. (doi: 10.3390/jcm13020382) PMC10816178 38256519

[20] Caroppo E Castiglioni F Cabrilo K Bellavia M Gazzano G Colpi GM. Oligoasthenoteratozoospermic patients with high untreatable sperm DNA fragmentation and prior ICSI failure using ejaculated sperm have signs of partial epididymal obstruction. Andrology. Published online April 12, 2025. (doi: 10.1111/andr.70044) 40220323

[21] Colpi G Pinggera GM Calogero A Merit-based academic certification in andrology: a groundbreaking initiative by the global andrology forum. World J Mens Health. Published online May 12, 2025. (doi: 10.5534/wjmh.250038) PMC13302991 40583023

